# Viral Inhibition of the Transporter Associated with Antigen Processing (TAP): A Striking Example of Functional Convergent Evolution

**DOI:** 10.1371/journal.ppat.1004743

**Published:** 2015-04-16

**Authors:** Marieke C. Verweij, Daniëlle Horst, Bryan D. Griffin, Rutger D. Luteijn, Andrew J. Davison, Maaike E. Ressing, Emmanuel J. H. J. Wiertz

**Affiliations:** 1 Department of Medical Microbiology, University Medical Center Utrecht, Utrecht, The Netherlands; 2 MRC—University of Glasgow Centre for Virus Research, Glasgow, United Kingdom; University of Notre Dame, UNITED STATES

## Abstract

Herpesviruses are large DNA viruses that are highly abundant within their host populations. Even in the presence of a healthy immune system, these viruses manage to cause lifelong infections. This persistence is partially mediated by the virus entering latency, a phase of infection characterized by limited viral protein expression. Moreover, herpesviruses have devoted a significant part of their coding capacity to immune evasion strategies. It is believed that the close coexistence of herpesviruses and their hosts has resulted in the evolution of viral proteins that specifically attack multiple arms of the host immune system. Cytotoxic T lymphocytes (CTLs) play an important role in antiviral immunity. CTLs recognize their target through viral peptides presented in the context of MHC molecules at the cell surface. Every herpesvirus studied to date encodes multiple immune evasion molecules that effectively interfere with specific steps of the MHC class I antigen presentation pathway. The transporter associated with antigen processing (TAP) plays a key role in the loading of viral peptides onto MHC class I molecules. This is reflected by the numerous ways herpesviruses have developed to block TAP function. In this review, we describe the characteristics and mechanisms of action of all known virus-encoded TAP inhibitors. Orthologs of these proteins encoded by related viruses are identified, and the conservation of TAP inhibition is discussed. A phylogenetic analysis of members of the family Herpesviridae is included to study the origin of these molecules. In addition, we discuss the characteristics of the first TAP inhibitor identified outside the herpesvirus family, namely, in cowpox virus. The strategies of TAP inhibition employed by viruses are very distinct and are likely to have been acquired independently during evolution. These findings and the recent discovery of a non-herpesvirus TAP inhibitor represent a striking example of functional convergent evolution.

## Introduction

The family Herpesviridae emerged approximately 400 million years ago [1]. The first members of the class Mammalia arose 200 million years ago, at around the time of the Early Jurassic period, and, since then, herpesviruses and mammals have coevolved and adapted to one another over very long periods of time. Today, members of the family Herpesviridae are numerous and widespread among not only mammals, but also many bird and reptile species; each virus displays a remarkable degree of host specificity. The longstanding interactions between virus and host have likely contributed to the development of the host’s innate and adaptive immune system and the mechanisms that viruses use to evade those systems.

The first line of defense against intruding pathogens is the innate immune system. This comprises the complement system, natural killer (NK) cells, apoptosis, pattern recognition receptor-mediated intracellular signaling leading to the production of IFNβ and many other cytokines and chemokines, and phagocytes like neutrophils, macrophages and dendritic cells. Together, these mechanisms enable the host to limit replication and spread of a pathogen and facilitate the induction of specific adaptive immune responses. The adaptive immune system includes antibody-producing B-cells, CD4^+^ T-cells that recognize antigens presented in the context of MHC (class) II molecules, and CD8^+^ T-cells that generally recognize antigens in the context of MHC I molecules.

During and following protein synthesis, a proportion of the resulting proteins is rapidly degraded into peptides by the proteasome. The resulting peptides are subsequently translocated into the lumen of the endoplasmic reticulum (ER) via the transporter associated with antigen processing (TAP) [2,3]. Within the ER, the peptides are loaded onto newly synthesized MHC I heavy chain / β_2_microglobulin (β_2_m) heterodimers. This process is facilitated by at least five ER-resident molecules that together form the MHC I peptide-loading complex (PLC). Tapasin functions as a chaperone, bridging MHC I molecules and TAP and catalyzing the binding of high-affinity peptides [4–10]. The lectin-like chaperones calnexin and calreticulin promote folding of newly synthesized MHC I molecules; additionally, calreticulin recruits the thioloxidoreductase ERp57. ERp57 and protein disulfide isomerase (PDI) are involved in stabilizing several protein-protein interactions within the PLC via disulfide bond formation [11,12]. Acquisition of peptide allows mature MHC I complexes to leave the ER, pass through the Golgi, and traffic to the cell surface where the peptides are presented to CTLs.

The family Herpesviridae is divided into the subfamilies Alphaherpesvirinae, Betaherpesvirinae, and Gammaherpesvirinae. Members of this family have been identified in many different species, including reptiles, birds, and mammals. There are nine herpesviruses known to infect humans: herpes simplex virus (HSV) types 1 and 2 (HSV-1 and HSV-2 in species *Human herpesvirus 1* and *Human herpesvirus 2*, respectively, of genus *Simplexvirus*, subfamily Alphaherpesvirinae), varicella-zoster virus (VZV in species *Human herpesvirus 3* of genus *Varicellovirus*, subfamily Alphaherpesvirinae), human cytomegalovirus (HCMV in species *Human herpesvirus 5* of genus *Cytomegalovirus*, subfamily Betaherpesvirinae), human herpesviruses 6A, 6B and 7 (HHV-6A, HHV-6B, HHV-7 in species *Human herpesvirus 6A*, *Human herpesvirus 6B* and *Human herpesvirus 7* of genus *Roseolovirus*, subfamily Betaherpesvirinae), Epstein-Barr virus (EBV in species *Human herpesvirus 4* of genus *Lymphocryptovirus*, subfamily Gammaherpesvirinae) and Kaposi’s sarcoma-associated herpesvirus (KSHV in species *Human herpesvirus 8* of genus *Rhadinovirus*, subfamily Gammaherpesvirinae) [13]. Most of these viruses are widespread within the human population; for example, in the United States, approximately 90% of individuals of 80 years or older are seropositive for HCMV [14]. VZV is even more abundant, with a seroprevalence of 95% in people from 20 years of age [15]. Herpesvirus infections generally cause only mild symptoms, but in some circumstances they exhibit significant pathogenic properties, with the most serious complications tending to arise in immunocompromised people. Thus, HSV-1 can cause encephalitis, EBV and KSHV are associated with malignancies, and HCMV infection can result in congenital defects [16–18].

Following primary infection of their host, herpesviruses establish a state of latency in which viral protein expression is limited. As a consequence of this strategy, the virus-derived pool of potential antigens is minimized, thus hindering recognition and elimination of infected cells by CTLs and enabling the virus to persist for the lifetime of the host. In addition, several latency-associated proteins have been found actively to impede detection of virus-infected cells by the host immune system [19–21]. However, at some point, in order to disseminate the virus to other hosts, productive infection must occur. During this replicative or lytic phase, an extensive repertoire of herpesvirus-encoded genes is expressed in a kinetically regulated fashion. Depending on the virus in question, this results in the synthesis of at least 70 functional proteins, and renders the virus-infected cell susceptible to the memory immune responses that were generated during primary infection. Although these responses are instrumental in controlling infection and in limiting pathology, herpesviruses employ multiple evasion strategies to allow virus production in the face of existing antiviral immunity, thereby promoting spread within the host population.

In the past two decades, numerous articles have been published identifying and characterizing the immune evasion molecules expressed by herpesviruses. Most of these publications are focused on human herpesviruses, each of which has been shown to employ several strategies to interfere with the innate and adaptive immune responses. The MHC I presentation pathway appears to be a favorite target among the herpesviruses, illustrating the importance of CTLs in the elimination of virus-infected cells. Every step of this pathway is targeted by at least one herpesvirus. The availability of MHC I is affected by so-called host shutoff proteins that block cellular protein synthesis and thus expression of newly synthesized MHC I molecules. Examples of these proteins are the HSV-1-encoded vhs or UL41, EBV BGLF5, and KSHV SOX or ORF37 [22–26]. Additional strategies focus on inducing the degradation of MHC I molecules, as mediated by HCMV US2, US10, and US11 [27–29], murine CMV (MCMV) glycoprotein (gp) 48 [30], and murine gammaherpesvirus 68 mK3 [31–33]. Presentation of antigenic peptides by MHC I is also affected by HCMV US3 and MCMV gp40, which cause the retention of immature molecules in the *cis*-Golgi [34,35]. Finally, EBV BILF1 and KSHV K3 and KSHV K5 enhance the endocytosis of MHC I complexes at the cell surface [36–39].

**Fig 1:**
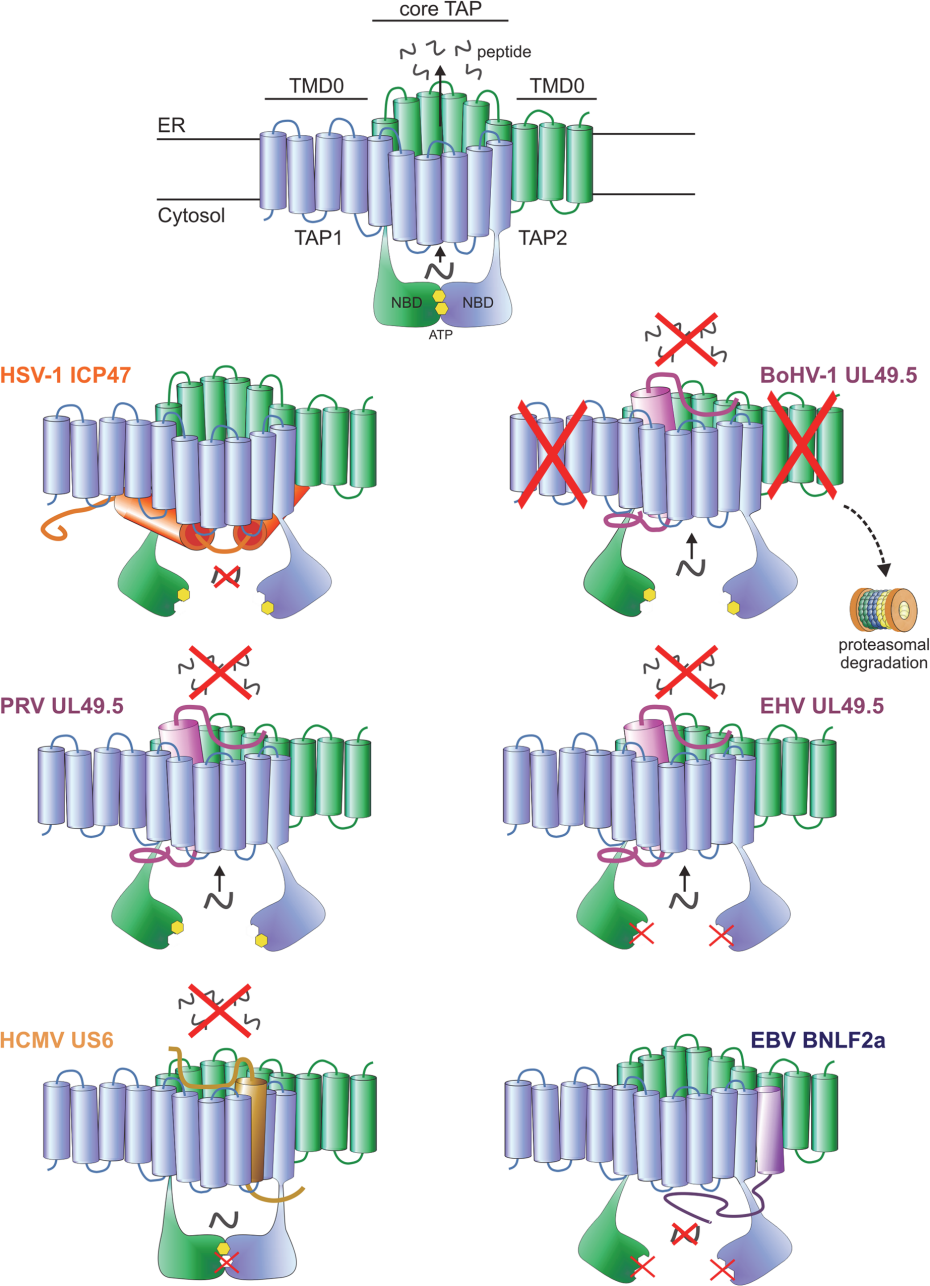
Interactions between herpesvirus-encoded TAP-inhibitors and their target. Upper illustration: model of the TAP transporter, comprising the two subunits TAP1 and TAP2. Each subunit contains a transmembrane domain (TMD), encompassing 10 and 9 transmembrane (TM) helices for TAP1 and TAP2, respectively. The outer N-terminal helices of TAP1 and TAP2 (TMD0) form an autonomous binding platform for tapasin, whereas the core 6 TM helices are necessary for peptide transport. A peptide-binding domain is located within the cytosolic extensions of the TM helices. In addition, TAP1 and TAP2 contain a nucleotide-binding domain (NBD) in the cytosol, which harbors two ATP-binding sites. Lower illustrations: schematic representations of the interaction between the viral proteins and TAP. The sites where TAP is affected are indicated. HSV-1 ICP47 prevents peptide transport by physically obstructing the peptide-binding site. PRV, BoHV-1 and EHV (EHV-1 and EHV-4) UL49.5 leave the transporter in a transformation-incompetent conformation, thereby preventing the structural changes that are needed to translocate peptides over the ER membrane. BoHV-1 UL49.5 is known to interact with a region within the core domain of TAP, comprising the C-terminal 6 TM domains of both TAP1 and TAP2 [132]. BoHV-1 UL49.5 induces the degradation of both TAP subunits, and EHV UL49.5 prevents ATP binding to TAP. HCMV US6 blocks TAP by inducing conformational changes that result in diminished ATP binding to TAP1. The protein interacts with TM domains 7–10 of TAP 1 and TM 1–4 of TAP2 [95]. EBV BNLF2a inhibits peptide transport by interfering with both peptide and ATP binding to TAP.

In addition to strategies that limit the availability of MHC I, many herpesviruses affect peptide presentation by inhibiting the function of TAP. TAP is a heterodimeric ATP-binding cassette (ABC) transporter complex composed of two subunits, TAP1 and TAP2 (Fig 1). Both subunits are composed of an N-terminal transmembrane domain (TMD) and a C-terminal nucleotide-binding domain (NBD) exposed in the cytosol (Fig 1). The TMDs of TAP1 and TAP2 contain 10 and 9 transmembrane (TM) helices, respectively. The N-terminal 4 TM helices of TAP1 and the N-terminal 3 TM helices TAP2, known as TMD0s, act as autonomous interaction platforms for tapasin [40]. Together with the NBDs, the C-terminal 6 membrane helices of each TAP subunit form the core of the transporter. Expression of this core region is necessary and sufficient for peptide transport [41].

TAP preferentially transports peptides of 8–16 amino acid residues in length, but can accommodate peptides as large as 40 amino acid residues, albeit with lower efficiency [42–46]. The exact location of the peptide-binding pocket remains unclear, but cross-linking peptide substrates to TAP and mutagenesis of TAP have shown that elements within the cytosolic extensions between the TM helices of both TAP1 and TAP2 are involved in peptide binding to TAP [47,48]. These findings are further supported by homology modeling of TAP based on the resolved crystal structures of other ABC transporters [49]. Three cytosolic pockets formed by TM helices of the TAP core complex have been proposed to represent the binding site for the peptide substrates [49].

TAP-mediated peptide transport is energized by ATP hydrolysis at the NBDs, which harbor two functionally nonequivalent ATP-binding sites. These sites are composed of conserved domains within both subunits, including the Walker A and B domains of one NBD and a signature motif of the opposing NBD. The consensus ATP-binding site that includes the Walker A and B domains of TAP2 has catalytic amino acid residues conserved among ABC transporters. The degenerate ATP-binding site, which includes the Walker A and B domains of TAP1, has a number of noncanonical mutations that reduce its catalytic activity [50]. Although ATP binding and hydrolysis can still occur at the degenerate site [51,52] only ATP binding and hydrolysis at the consensus TAP2 site is essential for completion of the transport cycle [50,52–54].

The crystal structures of several ABC transporters, trapped in distinct conformations, have been resolved [55–59]. These structures, together with biochemical studies on TAP itself, suggest that TAP transport occurs in sequential steps with extensive conformational rearrangements of the NBDs and TMDs, which depend on nucleotide and peptide substrate binding [60,61] (reviewed by [62]). In an inward-facing conformation, the peptide-binding pocket faces the cytosol and the NBDs are separated. At this stage, TAP is receptive to both peptide and ATP. The binding of peptide and ATP can occur independently and induces conformational rearrangements that partially close the NBDs. The NBDs are only fully closed when both peptide and ATP are bound. These conformational changes are relayed to the TMDs and result in an outward-facing conformation of TAP, thereby exposing the peptide-binding pocket into the ER lumen and allowing release of the peptide. Upon ATP hydrolysis, the NBDs dissociate and the TMDs rotate back into an inward-facing conformation [62]. In this way, conformational changes driven by peptide binding, ADP/ATP exchange, and ATP hydrolysis at the TAP subunits lead to the transport of peptides into the ER lumen.

Cells that naturally or experimentally lack expression of functional TAP complexes show a dramatic reduction in MHC I levels at their surface and a substantial decline in CTL sensitivity [63–68]. Herpesviruses appear to have taken advantage of this extensive dependency of MHC I expression on TAP function by encoding viral proteins that specifically impair TAP-mediated peptide transport. This review focuses on the characteristics and evolution of herpesvirus-encoded TAP inhibitors and their orthologs.

### Simplexvirus ICP47 Orthologs

The first viral protein found to inhibit TAP function was HSV-1 ICP47 (Fig 1). This protein acts as a competitor of cytosolic peptides for TAP binding, thereby limiting the availability of peptides in the ER lumen and causing subsequent retention of MHC I molecules in the ER [69–72]. ICP47 is expressed as a soluble, cytosolic protein of 88 amino acid residues. Mutational analyses have defined residues 3–34 as the functional domain responsible for TAP binding and inhibition of TAP function [73–75]. Charged residues within this domain are crucial for TAP inhibition, and might mimic the N- and C-termini of peptide substrates within the peptide-binding pockets of TAP [75]. Once bound to TAP, the viral protein traps TAP in a conformation that differs from that of TAP in a peptide-bound state [76]. In contrast to peptide substrates, ICP47 blocks ATP hydrolysis at the NBDs of TAP, which is normally followed by peptide binding, suggesting that TAP is locked in an inward-facing conformation [51,77].

**Fig 2:**
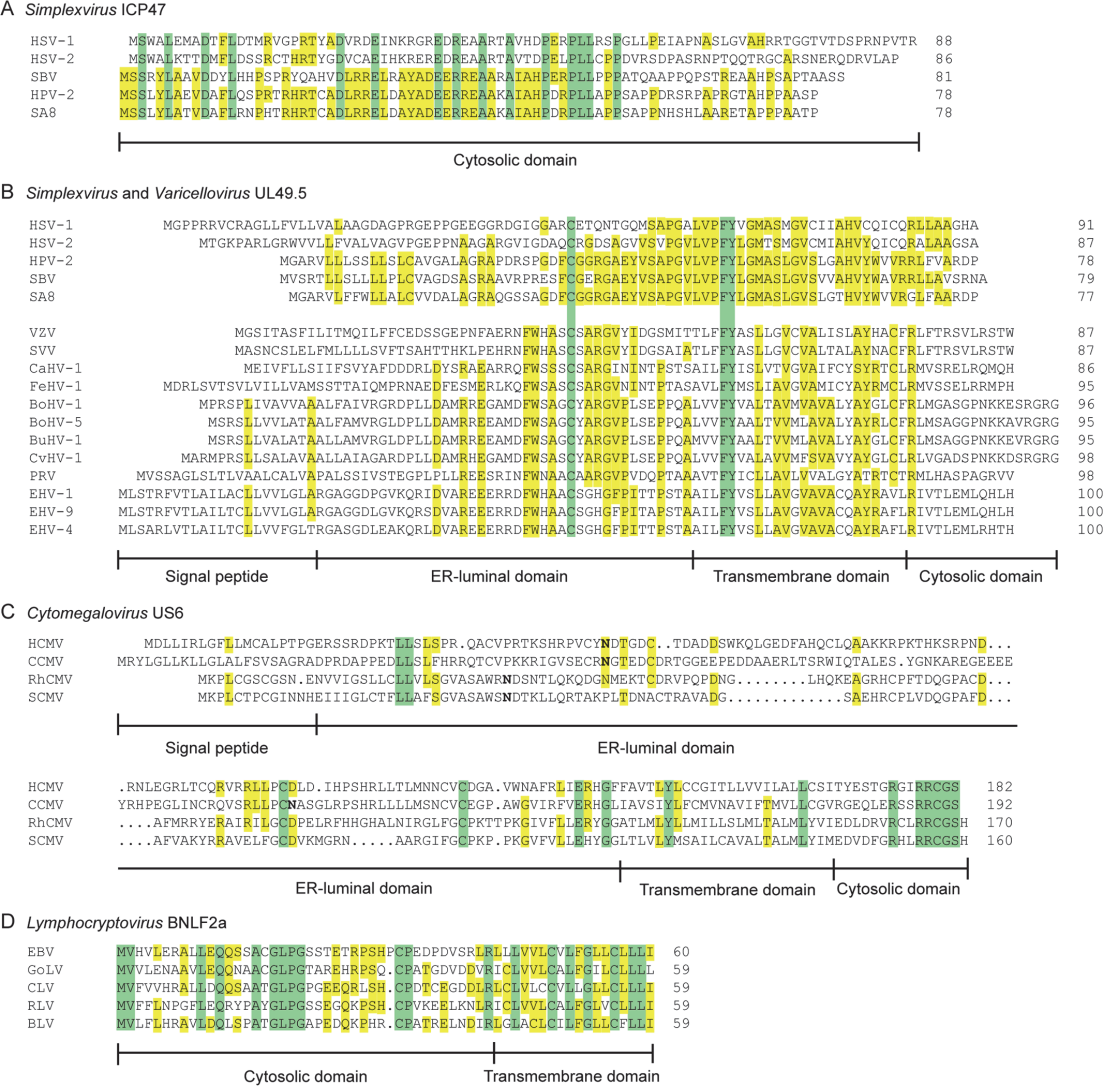
Alignments of the amino acid sequences of selected herpesvirus-encoded TAP-inhibitors. A) simplexvirus ICP47 orthologs, B) simplexvirus (upper 5 lines) and varicellovirus (lower 12 lines) UL49.5 orthologs, C) cytomegalovirus US6 orthologs, and D) lymphocryptovirus BNLF2a orthologs. The alignments of predicted primary translation products were made using ClustalW, followed by manual adjustment. The number of residues in each sequence is shown on the right. Green highlights residues that are conserved in all sequences, and yellow highlights residues that are conserved in a majority. Bold N residues in US6 indicate potential N-linked glycosylation sites. An illustrationof sequence disposition is shown below each alignment, with approximate boundaries displayed.

**Fig 3:**
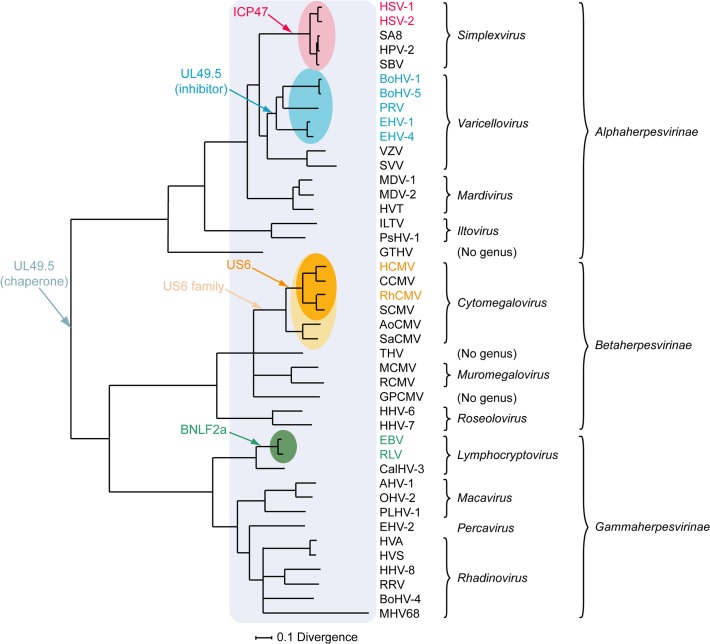
Phylogenetic tree for selected members of the family Herpesviridae. The Bayesian tree is based on amino acid sequence alignments for six large, well-conserved genes, namely the orthologs of HSV-1 genes UL15, UL19, UL27, UL28, UL29, and UL30, and is derived from McGeoch and Davison [81]. Assignments to genera and subfamilies are shown on the right. Abbreviations not mentioned in the text are: MDV-1, Marek's disease virus type 1; MDV-2, Marek's disease virus type 2; HVT, herpesvirus of turkey; ILTV, infectious laryngotracheitis virus; PsHV-1, psitticid herpesvirus 1; GTHV, green turtle herpesvirus; THV, tupaia herpesvirus; GPCMV, guinea pig cytomegalovirus; CalHV-3, callitrichine herpesvirus 3; AHV-1, alcelaphine herpesvirus 1; OHV-2, ovine herpesvirus 2; PLHV-1, porcine lymphotropic herpesvirus 1; HVS, herpesvirus saimiri; HVA, herpesvirus ateles; and RRV, rhesus rhadinovirus. Red, blue, orange, and green shading indicate viruses that encode the ICP47, UL49.5, US6, or BNLF2a TAP inhibitor genes, respectively, and corresponding coloring of virus abbreviations indicate viruses in which these genes have been shown to be functional TAP inhibitors. Light orange shading identifies all members of the *Cytomegalovirus* genus that have a US6 gene. Light blue shading indicates all members of the herpesvirus family that code for a UL49.5 gene that might be involved in chaperoning maturation of glycoprotein M.

Orthologs of HSV-1 ICP47 are encoded by HSV-2 and other simplexviruses infecting Old World primates, including herpesvirus papio 2 (HVP-2 in species *Papiine herpesvirus 2*) for baboons, simian B virus (SBV in species *Macacine herpesvirus 1*) for macaques, and simian agent 8 (SA8 in species *Cercopithecine herpesvirus 2*) for African green monkeys [78]. Inhibition of TAP is conserved for HSV-2 ICP47, despite the relatively low amino acid sequence identity (44%) (Fig 2A) [79]. The ICP47 orthologs can be divided into two groups on the basis of sequence similarities within the domain responsible for TAP inhibition, i.e., amino acid residues 3 to 34 [73–75]. HSV-1 and HSV-2 ICP47 are in the first group and share identity within this domain, and both are known to inhibit TAP (Fig 2A) [79]. HPV-2, SBV, and SA8 ICP47 are in the second group. The sequences of the N-terminal domain of these proteins show substantial identity to each other and differ substantially from those of HSV-1 and HSV-2 (Fig 2A). SBV-infected cells were shown to display minor MHC I downregulation compared to HSV-1-infected cells [80], suggesting that the ICP47 protein encoded by SBV does not inhibit TAP. Given the similarities between the SBV, HPV-2, and SA8 ICP47 proteins, the HPV-2 and SA8 ICP47 proteins may also be unable to inhibit TAP, but data are not available. Thus, TAP inhibition may not be conserved for all ICP47 proteins. The fact that ICP47 is only encoded by simplexviruses suggests that this gene evolved after this lineage separated from the varicelloviruses (Fig 3). There is evidence supporting the view that the ICP47 gene arose de novo, rather than being captured from elsewhere, as is the case for many immune modulatory genes in herpesviruses [81].

### Varicellovirus UL49.5 Orthologs

The second TAP inhibitor discovered within the subfamily Alphaherpesvirinae is the varicellovirus UL49.5 protein. The most extensively studied member is bovine herpesvirus 1 (BoHV-1) UL49.5. Unlike ICP47, UL49.5 does not affect the binding of peptides to TAP. Instead, the viral protein binds to the core region of TAP and impairs TAP-mediated peptide transport through two unique mechanisms. First, BoHV-1 UL49.5 inhibits the conformational rearrangements that usually follow peptide and ATP binding, as inferred from fluorescence recovery after photo bleaching (FRAP) assays [82]. Using this assay, the lateral mobility of Green Fluorescent Protein (GFP)-tagged TAP can be measured in the ER-membrane. TAP mobility is affected by the conformational rearrangements that occur upon peptide transport. Upon active peptide transport, the mobility of TAP molecules is slower than that of inactive TAP [2]. In the presence of UL49.5, these changes in lateral mobility of TAP, and thus conformational rearrangements, are halted [82]. Second, BoHV-1 UL49.5 strongly reduces TAP1 and TAP2 protein levels by targeting both TAP subunits for proteasomal degradation (Fig 1) [82]. Mutational analysis of BoHV-1 UL49.5 has attributed degradation of TAP to the first five N-terminal amino acid residues and the ultimate residues of the C-terminal cytosolic domain of the protein [83]. Interestingly, UL49.5 without a C-terminal domain does not cause degradation of TAP, but retains the ability to inhibit peptide transport; this implies that the regions responsible for this effect are located in the transmembrane or ER-luminal parts of the inhibitor [82].

Orthologs of UL49.5, also known as glycoprotein N (gN), are present in all members of the family Herpesviridae sequenced to date. However, TAP inhibition by this protein has been found only among the varicelloviruses. The UL49.5 orthologs encoded by the varicelloviruses BoHV-5, bubaline herpesvirus 1 (BuHV-1), cervid herpesvirus 1 (CvHV-1), equid herpesvirus 1 (EHV-1), EHV-4, pseudorabies virus (PRV), and felid herpesvirus 1 (FeHV-1) possess the same functional properties as BoHV-1 UL49.5, causing a robust inhibition of peptide transport, thereby decreasing MHC I molecules at the cell surface [84,85]. Infections with UL49.5-deletion mutants of BoHV-1, EHV-1, and PRV have shown that UL49.5 is necessary and sufficient for TAP inhibition during viral infection in vitro [84]. Surprisingly, UL49.5 expressed by the varicelloviruses VZV, simian varicella virus (SVV), and canid herpesvirus 1 (CaHV-1) are incapable of reducing TAP function [85]. Thus, the capacity to interfere with peptide transport via TAP is a feature shared by a subgroup of varicellovirus-encoded UL49.5 orthologs.

The TAP-inhibiting UL49.5 homologs were shown to block human TAP as well as their natural host TAPs, indicating that the proteins target a highly conserved region within the TAP complex [84,85]. Each UL49.5 ortholog appears to utilize a distinct mechanism of TAP inhibition. The capacity to arrest the TAP complex in a translocation incompetent state is conserved for EHV-1 and PRV UL49.5 (Fig 1) [84] and most likely for EHV-4, BoHV-5, BuHV-1, CvHV-1, and FeHV-1 UL49.5 [85]. The EHV-1 and EHV-4 UL49.5 proteins were shown to interfere uniquely with ATP binding to TAP (Fig 1) [84]. UL49.5-induced degradation of TAP1 and TAP2 is conserved for the highly related viruses BoHV-1, BoHV-5, BuHV-1, and CvHV-1, all of which infect ruminants, but not for EHV-1, EHV-4, PRV, and FeHV-1 UL49.5 (Fig 1) [85]. The UL49.5 proteins interfering with TAP function show around 40% sequence identity (Fig 2B). Proteasomal degradation of the transporter is only induced by the ruminant-infecting viruses. The cytoplasmic domain of the TAP-degrading UL49.5 proteins contains two unique, consecutive lysine residues and an RGRG motif (Fig 2B). These lysine residues, although potential targets for ubiquitination, are not required for degradation of TAP [83]. However, the arginine residues of the RGRG sequence appeared to be essential for this phenotype [83].

Based on current knowledge, the UL49.5 molecules are the only herpesvirus-encoded TAP inhibitors that fulfill a dual role in viral infection. Within the infected cell, UL49.5 forms a heterodimeric complex with glycoprotein M (gM) and guides proper glycosylation and maturation of this protein [86–88]. Cells expressing both BoHV-1 UL49.5 and gM show reduced TAP inhibition when compared to cells expressing UL49.5 only, suggesting that the interaction between UL49.5 and gM interferes with the capacity of UL49.5 to block TAP [89]. However, UL49.5 and gM display differential temporal expression in the context of viral infection, with the appearance of UL49.5 preceding that of the late protein gM [89]. This provides UL49.5 with an opportunity to exert its immune evasive effect early during infection. Conservation of both UL49.5 and gM in the family Herpesviridae indicates that the original role of UL49.5 was that of a gM chaperone, and that TAP inhibition by the protein evolved later within the varicellovirus subfamily, possibly in the BoHV-1 lineage after its divergence from the VZV lineage (Fig 3). Alternatively, it is possible that the TAP inhibitory function was gained somewhat earlier among the alphaherpesviruses, and lost in the VZV lineage, as VZV UL49.5 is capable of interacting with the TAP complex even though it does not inhibit its activity.

### Cytomegalovirus US6 Orthologs

HCMV encodes multiple immunoevasive proteins targeting the MHC I antigen presentation pathway, including the TAP inhibitor US6 [90–92]. This protein impairs TAP function by interfering with ATP binding to the transporter (Fig 1). US6 specifically prevents ATP binding to TAP1, but stimulates ATP binding to TAP2 [93]. This is in contrast with the normal pattern of ATP binding, which preferentially involves TAP1 [94]. US6 alters ATP binding to TAP by inducing conformational rearrangements that are suggested to resemble TAP in an outward-facing conformation [77,95]. Rather than physically obstructing the ATP-binding site in the cytosol, US6 induces these rearrangements by interacting with the ER luminal loops of TAP1 and TAP2 [95]. This observation is supported by data obtained with US6 truncation mutants, which shows that the ER luminal domain of US6 is necessary and sufficient for the inhibition of TAP [90,93].

Orthologs of US6 are only encoded by cytomegaloviruses infecting primates [96]. The rhesus CMV (RhCMV) ortholog of US6 (Rh185) shares only about 23% amino acid identity but nevertheless reduces cell surface expression of MHC I molecules via TAP inhibition (Fig 2C). The ER-luminal domain, identified as the functional part of US6 [90,93], shows remarkably low identity between the two orthologs (Fig 2C). HCMV US6 is a member of the US6 gene family, which is presumed to have arisen through gene duplication of a captured gene. The original gene gave rise to a block of six contiguous paralogs (US6, US7, US8, US9, US10, and US11), all encoding loosely related type I membrane proteins. Both the number of genes in this family and their encoded sequences have diverged extensively among the primate cytomegaloviruses. For example, chimpanzee cytomegalovirus (CCMV) and HCMV both encode six homologous genes in the US6 family. In contrast, RhCMV and simian CMV (SCMV) each have five genes, with orthology to HCMV being more difficult to determine [97]. Owl monkey cytomegalovirus (AoCMV) and squirrel monkey cytomegalovirus (SaCMV), which infect New World primates, have four and seven members of the US6 family, respectively. However, these are located in noncontiguous regions of the genome, and both viruses lack an obvious US6 ortholog. No US6 family genes are apparent in cytomegaloviruses of non-primate hosts, including MCMV and rat CMV (RCMV) [96]. Thus, it seems that the US6 gene family probably evolved during early primate evolution, with the TAP-inhibiting function arising in the Old World primate lineage (Fig 3).

### Lymphocryptovirus BNLF2a Orthologs

The gammaherpesvirus EBV codes for a lytically expressed TAP inhibitor, BNLF2a, that inhibits TAP by interfering with the binding of peptides and ATP to the transporter (Fig 1) [98,99]. Mechanistically, BNLF2a is thought to induce conformational changes in the TAP complex that prevent association of ATP and peptide, but the sequence of events preceding the block in TAP function remains to be elucidated. BNLF2a consists of a hydrophilic N-terminal domain and a hydrophobic C-terminal domain (Fig 2D). BNLF2a lacks an obvious N-terminal signal sequence but is membrane-integrated, nevertheless. The protein was identified as a tail-anchored transmembrane protein that uses Asna1/TRC40, among other proteins, for ER membrane insertion [100]. This mechanism of localizing to membranes is unique among the known viral TAP inhibitors.

Orthologs of BNLF2a have only been identified in lymphocryptoviruses that infect Old World primates [98]. The BNLF2a orthologs encoded by rhesus, chimpanzee, baboon, and gorilla lymphocryptoviruses (RLV, CLV, BLV, and GoLV) share 53%–62% sequence identity with EBV BNLF2a and display a similar disposition of hydrophilic and hydrophobic regions (Fig 2D). When expressed in isolation, these orthologs downregulate cell surface expression of MHC I molecules, indicating conserved TAP-inhibiting properties for BNLF2a proteins expressed by lymphocryptoviruses of Old World primates [98]. No orthologs of BNLF2a have been detected in members of the genus *Lymphocryptovirus* that infect New World primates, suggesting that the BNLF2a gene was acquired after the divergence of Old World and New World primate lymphocryptoviruses (Fig 3).

## Conclusions

Members of all three subfamilies in the family Herpesviridae appear to exploit inhibition of TAP-mediated peptide transport as an immune evasion strategy. The TAP inhibitors that have been identified so far exhibit substantial variation in structural characteristics as well as in mechanisms of action. Yet, despite their large diversity, all inhibitors have evolved to serve a common end: diminish the supply of viral antigenic peptides into the ER lumen in order to avoid elimination of virus-infected cells by MHC I-restricted CTLs, thus ultimately aiding virus replication and spread. The manner in which this has been achieved represents a striking example of functional convergent evolution, and identifies TAP as an Achilles’ heel of the immune system.

All of the TAP-inhibiting proteins described in this review are expressed early during the viral replication cycle [89,91,99,101,102]. For example, EBV BNLF2a is expressed during the (immediate-) early phase of lytic replication, but the protein levels are reduced at later times in infection when other EBV-encoded immune evasive molecules are effective [103,104]. Similarly, BoHV-1 UL49.5 is expressed with early kinetics, but remains present during the late stage of infection [89]. This prolonged expression can be explained by the dual role that UL49.5 plays during viral replication: early in infection, in the absence of gM, it acts as a TAP inhibitor, while at later times of infection it also functions as a chaperone for the late, structural protein gM that facilitates cell-to-cell spread of virus [89]. The early expression of herpesvirus-encoded TAP inhibitors ensures inhibition of the transport of viral peptides into the ER for MHC association shortly after initiation of virus replication, before abundant viral protein synthesis starts. In support of this reasoning, T cell recognition of antigenic peptides expressed early after EBV reactivation is restored in cells infected with a BNLF2a-deleted recombinant EBV [103]. These findings further substantiate the contribution of the virus-encoded TAP inhibitors to immune evasion during infection.

Directly addressing the in vivo relevance of TAP inhibition for human herpesviruses is difficult, because of the restricted host specificity of these viruses. HSV is an exception to the rule, as this virus can productively infect mice. The functional relevance of ICP47 was addressed using a murine ocular infection model. Mice that received uniocular corneal infections with wild-type HSV-1 developed encephalitis and died within 12 days [105]. However, mice infected with an ICP47 deletion mutant did not develop encephalitis, pointing towards a role for ICP47 in preventing the activation of CD8^+^ T cells that avert the development of lethal encephalitis [105]. An additional study showed that in systemically infected mice, an ICP47 deletion mutant was also attenuated compared to wild type HSV-1. However, in mice lacking TAP, this phenotype was reverted in neuronal tissues, including the brain, suggesting that TAP inhibition is crucial for neuronal infection by HSV-1 [106]. The role of ICP47 in these mouse models seems contradictory to studies showing that ICP47 fails to inhibit TAP efficiently in mouse cell lines [69,70,79,107,108]. The low level of inhibition observed in vitro may be sufficient to generate a phenotype in vivo. Alternatively, ICP47 may block in vivo CD8^+^ T cell responses by additional mechanisms. In vivo studies on all other human herpesviruses depend on humanized mouse models. The identification of functional orthologs of, for example, US6 and BNLF2a in the genomes of Old World primate-infecting herpesviruses offers new opportunities to evaluate their contribution to replication and spread of the viruses in vivo. RhCMV, which encodes functional homologs of HCMV US2, US3, US6 and US11 [109], has been used to study the role of these immune evasion proteins in vivo [110]. RhCMV is known to be able to reinfect or superinfect its host, despite the presence of high levels of neutralizing antibodies and RhCMV-specific CD4^+^ and CD8^+^ T cells. However, rhesus macaques could not be superinfected by a RhCMV strain in which the genetic region encoding the US2, US3, US6, and US11 homologs was deleted. In contrast, the presence of these genes was not required for persistent infection of RhCMV-naïve hosts or for superinfection of macaques transiently depleted of CD8^+^ T cells. These studies indicate that impairment of MHC I presentation is critical for evading CD8^+^ T cell responses during superinfection by RhCMV, but not during primary infection [110].

The recent identification of the first viral TAP inhibitor outside the family Herpesviridae highlights the importance of targeting TAP function as a general viral immunoevasive strategy. Poxviruses, like herpesviruses, are known for their elaborate strategies aimed at evading the immune system. Cowpox virus (CPXV) is the first non-herpesvirus that has been found to encode a TAP inhibitor. This protein, CPXV012, is an ER-resident type II transmembrane protein of 69 amino acid residues that inhibits TAP through its ER-luminal domain by interfering with ATP binding to the NBDs of TAP [111–113]. A recent analysis of this gene in a range of CPXV strains has provided interesting clues as to the possible origin of the TAP-inhibiting capacity of CPXV012. It appears to have originated from a frameshifting deletion from a longer protein that possesses an extended ER-luminal region containing a C-type lectin-like domain [113,114]. The longer protein does not block TAP. The majority of the recently isolated clinical strains encode the shorter, TAP-inhibiting protein [113].

The identification of TAP inhibitors in herpesviruses and poxviruses suggests that DNA viruses in particular benefit from interference with TAP function as a means to evade CD8^+^ T cell responses. The absence of such evasion mechanisms in RNA viruses may in part be explained by the high mutation rate of RNA virus genomes, which allows for CD8^+^ T cell evasion by antigenic variation [115,116]. The generally lower mutation rate of DNA viruses may require alternative immune evasion mechanisms, including TAP inhibition. The relatively large genomes of herpes- and poxviruses have the capacity to accommodate dedicated immune evasion proteins that counteract the host immune response.

Apart from TAP, MHC I itself is also targeted directly by a wide range of viral proteins encoded by herpesviruses and poxviruses. Among the alphaherpesviruses, ORF66 of VZV binds to and accumulates MHC I in the Golgi compartment [117,118]. Several betaherpesviruses express proteins that induce degradation of MHC I via various pathways. US2, US10, and US11 of HCMV target MHC I for degradation via the ubiquitin-proteasome pathway [27–29]. MCMV-encoded gp48 and U21 of HHV-6 and HHV-7 mediate degradation of MHC I via the endolysosomal route [30,119,120]. The m152 protein of MCMV retains MHC I in intracellular compartments [35]. Rhesus CMV protein rh178 inhibits MHC I heavy chain expression by preventing its co-translational insertion into the ER membrane [121]. Also members of the gammaherpesvirus subfamily induce MHC I degradation using a variety of strategies. EBV-encoded BILF1 reroutes MHC I and targets it for lysosomal degradation [36,122]. MHV-68-encoded mK3 targets MHC I for proteasomal degradation [123], whereas kK3 and kk5 of KSHV degrade MHC I through the endolysosomal pathway [38].

In addition to herperviruses, poxviruses and adenoviruses also encode gene products that target MHC I directly. CPXV203 of cowpox virus and E3-19K of adenovirus both cause retention of MHC I in the ER [124–126].

The reduced overall MHC I surface expression induced by viral inhibitors makes cells more vulnerable for NK cell recognition. NK cells sense the lack of MHC I on target cells; this may result in a response depending on additional activation and inhibitory signals (reviewed by [127]). Herpes- and poxviruses use a variety of gene products to counteract NK cell responses. HCMV has been especially well studied for its ability to evade NK cell-mediated cytotoxicity (reviewed in [128]), but other herpesviruses and poxviruses also encode gene products to counteract NK cell-mediated cytotoxicity [129–131].

In conclusion, four classes of herpesvirus-encoded TAP inhibitors have been identified so far. These appear to be unrelated and to have been acquired independently and relatively recently during evolution, providing a powerful illustration of functional convergent evolution. In addition, poxvirus CPXV012 has been shown to code for yet another type of TAP inhibitor. Where in vitro and in vivo studies have been possible, they have demonstrated the significance of TAP inhibitors in the evasion of CTL recognition and replication of the virus in the face of potent immune responses. In addition to inhibition of TAP, MHC I function is inhibited by a large repertoire of unrelated viral gene products directly targeting MHC I, representing yet another example of functional convergent evolution. The acquisition of a wide range of unrelated proteins that interfere with MHC I-restricted antigen presentation highlights the importance of CTLs in antiviral immunity.
